# Time Trends in Stroke and Subtypes Mortality Attributable to Household Air Pollution in Chinese and Indian Adults: An Age-Period-Cohort Analysis Using the Global Burden of Disease Study 2019

**DOI:** 10.3389/fnagi.2022.740549

**Published:** 2022-02-18

**Authors:** Yudiyang Ma, Donghui Yang, Jianjun Bai, Yudi Zhao, Qian Hu, Chuanhua Yu

**Affiliations:** ^1^Department of Epidemiology and Biostatistics, School of Public Health, Wuhan University, Wuhan, China; ^2^Department of Public Health, Tongji Medical College, Huazhong University of Science and Technology, Wuhan, China

**Keywords:** stroke and subtypes, mortality, household air pollution, China, India, age-period-cohort (APC) analysis

## Abstract

Household air pollution (HAP) exposure is recognized as a major health concern in areas relied on residential burning of solid fuels for cooking and heating. However, previous study has focused on mortality across time and reported changes in age-specific mortality globally but failed to distinguish cohort from period effects. Therefore, this study aimed to differentiate the relative contributions of period and cohort effects to overall time trends of HAP-attributable stroke mortality between the most presentative East and South Asia countries. Data were obtained from the Global Burden of Disease (GBD) database. The age, period, and cohort effects were estimated using the age-period-cohort (APC) model. The overall age-standardized mortality rates (ASMRs) of stroke in China decreased by 39.8% compared with 35.8% in India, while stroke subtypes in both the sexes and countries showed consecutive significant declines from 1990 to 2019. The age-specific and cohort-specific HAP-attributable stroke mortality declined over time in China and India. By APC analysis, substantially increasing age effects were presented for stroke and subtypes from 25 to 84 years. China had a rapid reduction in the independent period and cohort effects. Also, the risk of death for subarachnoid hemorrhage (SAH) had the most striking decline for both sexes in period and cohort effects. Reductions of India were less favorable than China, but the independent period and cohort effects progressively decreased during the entire period for both the sexes. Males experienced a slightly higher mortality risk than females in both countries. Although prominent reductions were observed in HAP-attributable stroke and subtypes mortality during the past 30 years, China and India still suffered uneven HAP-attributable stroke burden. Thus, it is of high significance to introduce advanced solid fuels replace technology and knowledge regarding clean fuel use.

## Introduction

Household air pollution (HAP), which mainly refers to exposure to particulate matter with an aerodynamic diameter less than or equal to 2.5 μm (PM2.5), is recognized as a major health concern in areas relied on residential burning of solid fuels for cooking and heating (GBD 2016 Risk Factors Collaborators, 2018). Almost 3 billion people are counted on the traditional use of solid fuels to heat or cook globally. China and India still have approximately 32 and 55% of the population using solid fuels during everyday life (Cohen et al., 2017). Previous research studies denoted Asia leads the world in HAP-oriented deaths and HAP risk-oriented health problems have mainly occurred in low- and middle-income countries (LMICs) (Hystad et al., 2019; GBD 2019 Risk Factors Collaborators, 2020).

As one of the non-communicable diseases, stroke has become the second leading cause of deaths in 2019, which caused approximately 101 million cases and 6.55 million deaths worldwide (Roth et al., 2020). Stroke could be divided into three main pathologic types: ischemic stroke (IS), intracerebral hemorrhage (ICH), and subarachnoid hemorrhage (SAH) (Hisham and Bayraktutan, 2013). Each was caused by distinct underlying vascular pathologies. SAH poses a threaten on relatively young people, while IS and ICH are more common in middle-aged and elderly people (Feigin et al., 2005). Having more than one-third of the population of the world, China and India experience heavy personal, social, and financial costs of stroke, in the context of having a high burden stroke risk and rapidly evolving demography, lifestyles, and economies (Anderson and Chaturvedi, 2018). In 2019, stroke caused 2.2 million deaths in China, which accounted for 20.7% of total deaths and represented the first leading cause of deaths; whereas it caused 0.7 million deaths in India, accounting for 7.5% of total deaths and representing the third leading cause of deaths.

Household air pollution has been confirmed as one of the primary risk factors of stroke mortality in the countries heavily depending on solid fuels (Yu et al., 2018, 2020). A 10-μg/m^3^ increment in HAP was associated with 4–6% increase in overall mortality, 10% rise in cardiovascular disease prevalence, particularly among susceptible populations (Burnett et al., 2018; Xue et al., 2019). Over past years, a body of epidemiological research studies has reported that long-term HAP exposure was related to induction and progression of certain cardiovascular events. Likewise, it may escalate mortality of stroke, coronary artery disease, and acute myocardial infarction (Bruce et al., 2000; Balmes, 2019; Liu et al., 2021).

However, the previous study has focused on mortality across time and reported changes in age-specific mortality globally but failed to distinguish cohort from period effects (Lu et al., 2021). Meanwhile, there is no research clearly exploring the temporal trend of IS, ICH, and SAH. Therefore, we decomposed the independent effects of age, period, birth cohort by age-period-cohort (APC) model with an intrinsic estimator (IE) algorithm. Differentiating the relative contributions of period and cohort effects to overall time trends between the most presentative East and South Asia countries assists in determining the success of earlier policy interventions and identifying future targets. This study uses data between 1990 and 2019 from the GBD 2019 and concentrates on comparing the HAP-attributable stroke and subtypes mortality over the last three decades in China and India. This study could consolidate the body of evidence on existing disparities, changes, and hurdles in stroke research studies in LMICs.

## Materials and Methods

Contributed by the IHME, the GBD 2019 aims to quantify the comparative magnitude of health loss due to diseases, injuries, and risk factors by age, sex, and geographies for specific points in a series of time (GBD 2015 Mortality and Causes of Death Collaborators, 2016). All anonymized data have been publicly available at the website of the Institute for Health Metrics and Evaluation (IHME) and can be accessed online^1^. The informed consent was reviewed and approved by the University of Washington Institutional Review Board. It used DisMod-MR 2.1, a Bayesian meta-regression tool, as the primary method of estimation to ensure consistency between rates of incidence, prevalence, remission, and cause of deaths for each condition and provided a comprehensive annual estimation of global, regional, and national incidence, prevalence, mortality for causes of deaths and risk factors in 204 countries and territories from 1990 to 2019 (GBD 2019 Risk Factors Collaborators, 2020). The advantage of the GBD approach is that consistent methods are applied to critically appraise available information on each condition, making this information comparable and systematic; to estimate results from countries with incomplete data; and to report on the burden of disease with standardized metrics.

### Data Sources

The attributable burden of stroke data in China and India was obtained from the GBD 2019. Stroke was diagnosed and defined based on the WHO clinical criteria and the International Statistical Classification of Diseases. Stroke subtypes such as IS, ICH, and SCH events were classified using the tenth revision of the International Classification of Diseases and Injuries (ICD-10). The original data of stroke mortality in China population were mainly from the Cause of Deaths Reporting System of the Chinese Centers for Disease Control and Prevention (CDC) and Disease Surveillance Points (DSPs), which considered to be nationally representative (Zhou et al., 2016). Indian stroke mortality database was composed of vital registration (VR), verbal autopsy (VA), registry, survey, police, and surveillance data (Jha et al., 2006). The processed data was then to generate estimates of each quantity of interest by age, sex, location, and year. The modeling standardization was done using the Cause of Deaths Ensemble model (CODEm), spatiotemporal Gaussian process regression (ST-GPR), and DisMod-MR. The calculation formula for age-standardized mortality rate (ASMRs) is as follows:


$$\text{ASMRs}=\frac{\begin{array}{c} \sum A\,g\,e\,c\,o\,m\,p\,o\,s\,i\,t\,i\,o\,n\,o\,f\,s\,t\,a\,n\,d\,a\,r\,d\,g\,r\,o\,u\,p\,p\,o\,p\,u\,l\,a\,t\,i\,o\,n\\ \times A\,g\,e\,s\,p\,e\,c\,i\,f\,i\,c\,m\,o\,r\,t\,a\,l\,i\,t\,y \end{array}}{\begin{array}{c} A\,g\,e\,c\,o\,m\,p\,o\,s\,i\,t\,i\,o\,n\,o\,f\,s\,t\,a\,n\,d\,a\,r\,d\,p\,o\,p\,u\,l\,a\,t\,i\,o\,n \end{array}}$$

In the GBD study, HAP was defined as individual exposure to PM 2.5 due to the use of solid fuels and was assessed according to data of indoor solid fuel consumption and indoor PM 2.5 concentrations monitor (GBD 2016 Risk Factors Collaborators, 2018). Data were obtained from the standard multi-countries survey series such as Demographic and Health Surveys (DHS), Living Standards Measurement Surveys (LSMS), Multiple Indicator Cluster Surveys (MICS), and World Health Surveys (WHS), as well as country-specific survey series such as China Monitoring Survey and South Asia General Household Survey (GBD 2019 Risk Factors Collaborators, 2020). Population attributable fraction (PAF) was defined as if the exposure of a certain risk factor was reduced to the theoretical minimum exposure level in a certain population, the proportion of related diseases or deaths in the population would reduce (Burnett et al., 2014). In this study, the exposure level associated with minimum risk, known as the theoretical minimum risk exposure level, for HAP was between 2.4 and 5.9 μg/m^3^ (Yasin et al., 2019). The stroke mortality attributable to HAP was estimated based on defining PAF through combining the distribution of exposure to HAP with exposure-risk estimates at each level of exposure:


$$P\,A\,F=\frac{\sum_{i}^{n}P_{i}\,\left(R\,R_{i}-1\right)}{\sum_{i}^{n}P_{i}\,\left(R\,R_{i}-1\right)+1}$$

where *P*_*i*_ is the percentage of the population exposed to level *i* of HAP, *n* is the total number of exposure level. *RR*_*i*_ is the relative risk at exposure level *i*, which was estimated as the Integrated Exposure Response function of exposure based on 81 published systematic reviews, the specific methods are outlined in a previous study (GBD 2019 Risk Factors Collaborators, 2020).

Attributable deaths (ADs) were computed by multiplying the PAFs and the number of the deaths for stroke (*N*) (GBD 2017 Causes of Death Collaborators, 2018):


A⁢D=P⁢A⁢F*N

The age-standardized rate of HAP-ADs was calculated by the world standard population (Rudd et al., 2020).

Therefore, we obtained the population, mortality number, ASMRs data of HAP-attributable stroke, and the subtypes diseases in China and India from GBD 2019. Ethical approval was not needed for this study because there was no direct involvement of human subjects.

### Statistical Analyses

The APC model is a prevalently statistical model to extract information hidden in mortality which implies the death risks experienced by the population in a current year and the accumulation of health risks since birth (Cao et al., 2019). Typical statistical analysis could not decompose these death risks and health risks when estimating mortality. The APC model is used for long-term trend studies such as social changes, disease causes, aging, population process, and dynamic research (Yang et al., 2008). As the relationship between age, period, and cohort is perfectly linear (i.e., birth cohort = period-age), there is collinearity between age, period, and cohort (Robertson et al., 1999). Like previous studies, this study circumvents this problem by producing estimable APC parameters and functions without imposing arbitrary constraints on model parameters (Rosenberg and Anderson, 2011; Bell, 2020). Through the IE approach, the APC model performed by the freely available R-based tool from the US National Cancer Institute is designed to address the collinearity in APC model, and the detailed methodological information is described in former research (Rosenberg et al., 2014). The core operation code of R-based tool is accessible on GitHub.^2^

In a typical APC model, the age and period intervals must all be equal, i.e., 5-year age groups should be used with 5-year calendar periods. Therefore, the mortality and population data are arranged into 11 consecutive 5-year age groups from 25–29 (median, 27) to 75–79 years old (median, 82 years), six consecutive 5-year periods from 1990–1994 (median, 1992) to 2015–2019 (median, 2017), and 17 consecutive cohorts from born in 1910–1914 (median, 1912) to 1990–1994 (median, 1992). The reference groups in all the APC analyses are the central age group, period, or birth cohort in each interval. The fitted APC model estimates some useful parameters and functions in this study: (1) net drift is expressed as the annual percentage change of mortality, with the adjustments of the period and cohort effects; (2) local drift represents the average annual percentage changes in mortality over time across different age groups; (3) longitudinal age curve indicates the fitted longitudinal age-specific rates in the reference cohort adjusted for period deviations; (4) the period (or cohort) rate ratios (RRs) would be the relative risk adjusted for age and nonlinear effects in a period (or cohort) vs. the reference. For conducting APC analysis, the mortality and population data are arranged into consecutive 5-year periods from 1990 to 2019 and successive 5-year age intervals from 25–29 to 75–79 years old. The Wald chi-squared tests are adopted for estimation on the significance of the estimable functions. A general linear model is used to evaluate the interaction effect between sex and birth cohort or the significance of the slope of relative risks for period and cohort effect. *P <* 0.05 was considered as statistically significant. All the statistical tests are 2-sided.

## Results

### National Trends in Household Air Pollution-Attributable Stroke Mortality

**FIGURE 1:**
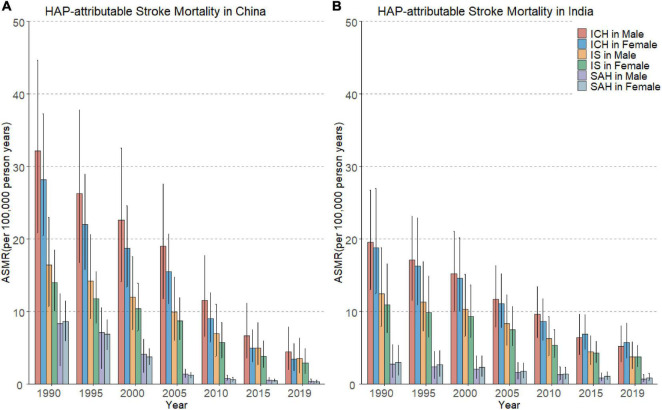
Trends in the age-standardized mortality rates (ASMRs) of household air pollution (HAP)-attributable stroke subtypes in China **(A)** and India **(B)** from 1990 to 2019. Error bars represented the 95% CIs for the HAP-attributable stroke mortality.

Figure 1 shows downward trends in HAP-attributable mortality across subtypes of stroke between China and India. From 1990 to 2019, the ASMRs of HAP-attributable stroke in China decreased by 39.8% compared with 35.8% in India. In 1990, the ASMRs of HAP-attributable ICH, IS, and SAH in Chinese (44.6 per 100,000, 16.4 per 100,000, and 8.3 per 100,000 in males; 28.1 per 100,000, 14.0 per 100,000, and 8.6 per 100,000 in females) were higher than its counterparts (19.6, 12.4, and 2.7 in males; 18.8, 10.9, and 3.0 in females) in India. However, in 2019, the ASMRs of these three subtypes (4.4, 3.5, and 0.4 in males or 3.4, 2.9, and 0.3 in females) in Chinese reduced to almost the same level as its counterparts (5.2, 3.7, and 0.7 in males or 5.7, 3.7, and 0.8 in females) in India. For subtypes of stroke, over the past 30 years, HAP-attributable ICH had the highest ASMRs among all the subtypes of stroke, followed by IS and SAH, and the sex disparity on the ASMRs shrunk with time.

### Age-Specific Mortality for Household Air Pollution-Attributable Stroke and Subtypes

Age-specific mortality of HAP-attributable stroke and subtypes by period across China and India are shown in Tables A1–A8, which presented the increased mortality with age group and a declining trend in mortality between 1910–1914 and 1990–1994 (*P <* 0.01 for all). The reduction of HAP-attributable ICH mortality was most marked for both countries. As shown in Supplementary Figure 1, China had a more obvious decreased trend of HAP-attributable stroke mortality across birth cohorts than India, indicating a relatively lower mortality risk in the cohort born recently in China (*P <* 0.01 for all).

### Age, Period, and Cohort Effects on Household Air Pollution-Attributable Stroke Mortality

**FIGURE 2:**
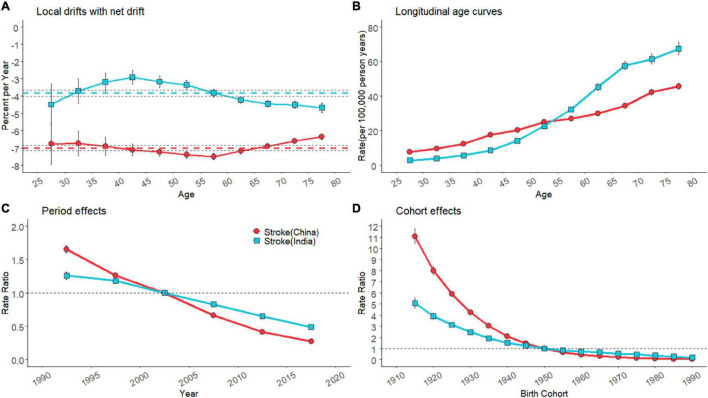
Parameter estimates of age, period, and cohort effects on HAP-attributable stroke mortality rate in China and India from 1990 to 2019. **(A)**, local drifts with net drifts for HAP-attributable stroke mortality. Net drifts were depicted as colored dashed lines with black dashed lines representing their 95% CIs. Error bars represented the 95% CIs for the local drift values (some were too narrow to show). **(B)**, fitted longitudinal age curves of HAP-attributable stroke mortality (per 100,000 person-years) and the corresponding 95% CIs. **(C)**, the rate ratios (RRs) of each period compared with the reference (2000–2004) adjusted for age and nonlinear cohort effects and the corresponding 95% CI. **(D)**, the RRs of each cohort compared with the reference (cohort 1950–1954) adjusted for age and nonlinear period effects and the corresponding 95% CI.

Substantial reductions in HAP-attributable stroke mortality were observed in the overall net drifts across the whole study period with a −7.0% (95% CI, −7.2 to −6.9%) change (Figure 2A). Mortality reductions were less evident in India [−3.8% (95% CI, −3.9 to −3.6%)]. Percent change per year (local drifts) was always below 0 in all the age groups for both the sexes and countries, indicating a decreasing trend in HAP-attributable stroke mortality across the study period (Figure 2A). For Indians, people aged over 40 experienced an accelerated reduction in mortality, whereas the greatest improvements on stroke were seen in Chinese aged 55–60 years (−7.5%/year). Age effects (Figure 2B) indicated upward trends in both the countries, with rates in India increasing rapidly in the later years of life (over 55 years old). Period and cohort effects tended to illustrate a monotonic decline in both countries. For India, the period effects had seen fewer improvements than China over the last 30 years (Figure 2C). The decline for HAP-attributable mortality appeared stagnant after the progressive improvement in Indians born before 1940 (Figure 2D).

### Age, Period, and Cohort Effects on Household Air Pollution-Attributable Stroke Subtypes Mortality

The local drifts and net drifts of stroke subtypes for males and females in China and India are shown in Table 1. All the local drifts and net drifts in both the countries were represented by negative values, which mean improvements in subtypes of stroke mortality. No matter China or India, the improvements were the most obvious in SAH, whereas modest in IS. Local drifts of these three subtypes in China indicated considerable improvements, especially in those aged over 50 years with SAH (<−11%/year for males and females). Even though the progress in controlling mortality of stroke subtypes in India was not striking as in China, mortality reductions in India were evenly in every subtype (around −3 to −5%/year).

**TABLE 1:** Local drifts and net drifts of household air pollution (HAP)-attributable subtype stroke for males and females in China and India.

Age	Local drift (95% CI)					
	ICH in male	ICH in female	IS in Male	IS in female	SAH in male	SAH in female
**China**						
25–29	−4.68 ± 1.06	−8.38 ± 2.49	−5.36 ± 1.18	−8.48 ± 2.47	−8.04 ± 1.28	−10.55 ± 2.20
30–34	−4.94 ± 0.63	−8.08 ± 1.47	−5.17 ± 0.69	−7.87 ± 1.49	−8.45 ± 0.85	−10.30 ± 1.37
35–39	−5.36 ± 0.45	−8.00 ± 1.02	−5.20 ± 0.46	−7.39 ± 1.02	−8.98 ± 0.62	−10.32 ± 0.96
40–44	−5.78 ± 0.31	−7.94 ± 0.66	−5.37 ± 0.29	−7.04 ± 0.66	−9.64 ± 0.44	−10.65 ± 0.64
45–49	−6.11 ± 0.24	−7.81 ± 0.48	−5.49 ± 0.20	−6.80 ± 0.45	−10.35 ± 0.35	−11.14 ± 0.49
50–54	−6.47 ± 0.19	−7.78 ± 0.39	−5.62 ± 0.15	−6.70 ± 0.34	−11.09 ± 0.30	−11.78 ± 0.41
55–59	−6.84 ± 0.18	−7.69 ± 0.34	−5.82 ± 0.12	−6.57 ± 0.28	−11.65 ± 0.27	−12.24 ± 0.38
60–64	−6.77 ± 0.15	−7.19 ± 0.29	−5.69 ± 0.09	−6.10 ± 0.21	−11.89 ± 0.24	−12.31 ± 0.32
65–69	−6.63 ± 0.14	−6.89 ± 0.36	−5.52 ± 0.08	−5.74 ± 0.16	−11.98 ± 0.23	−12.48 ± 0.31
70–74	−6.45 ± 0.15	−6.67 ± 0.27	−5.31 ± 0.08	−5.42 ± 0.15	−12.02 ± 0.24	−12.72 ± 0.32
75–79	−6.36 ± 0.19	−6.47 ± 0.32	−4.99 ± 0.10	−5.04 ± 0.18	−12.04 ± 0.29	−12.77 ± 0.37
Net Drift (95%CI)	−6.13 ± 0.15	−7.57 ± 0.33	−5.46 ± 0.14	−6.65 ± 0.32	−10.67 ± 0.21	−11.59 ± 0.32
**India**						
25–29	−4.12 ± 1.34	−5.11 ± 1.68	−3.43 ± 2.28	−4.29 ± 2.23	−4.17 ± 0.47	−4.44 ± 0.80
30–34	−3.30 ± 0.74	−4.17 ± 1.11	−2.77 ± 1.30	−3.36 ± 1.50	−3.75 ± 0.29	−3.90 ± 0.50
35–39	−2.95 ± 0.53	−3.32 ± 0.82	−2.48 ± 0.94	−2.52 ± 1.16	−3.54 ± 0.22	−3.47 ± 0.37
40–44	−2.82 ± 0.41	−2.81 ± 0.62	−2.34 ± 0.69	−1.91 ± 0.89	−3.55 ± 0.18	−3.30 ± 0.29
45–49	−3.21 ± 0.32	−2.97 ± 0.49	−2.62 ± 0.51	−1.94 ± 0.69	−3.91 ± 0.16	−3.55 ± 0.24
50–54	−3.57 ± 0.25	−3.01 ± 0.39	−2.90 ± 0.37	−1.94 ± 0.53	−4.24 ± 0.14	−3.74 ± 0.21
55–59	−4.06 ± 0.22	−3.66 ± 0.33	−3.31 ± 0.28	−2.63 ± 0.40	−4.59 ± 0.13	−4.16 ± 0.20
60–64	−4.60 ± 0.19	−4.04 ± 0.28	−3.91 ± 0.20	−3.12 ± 0.29	−4.93 ± 0.14	−4.44 ± 0.20
65–69	−4.92 ± 0.20	−4.27 ± 0.28	−4.28 ± 0.16	−3.56 ± 0.23	−5.16 ± 0.14	−4.67 ± 0.20
70–74	−4.94 ± 0.23	−4.47 ± 0.32	−4.34 ± 0.16	−3.87 ± 0.20	−5.30 ± 0.18	−4.92 ± 0.25
75–79	−5.07 ± 0.33	−4.80 ± 0.44	−4.49 ± 0.22	−4.23 ± 0.27	−5.51 ± 0.27	−5.24 ± 0.36
Net Drift (95% CI)	−3.88 ± 0.19	−3.71 ± 0.28	−3.27 ± 0.32	−2.83 ± 0.39	−4.37 ± 0.09	−4.07 ± 0.14

**FIGURE 3:**
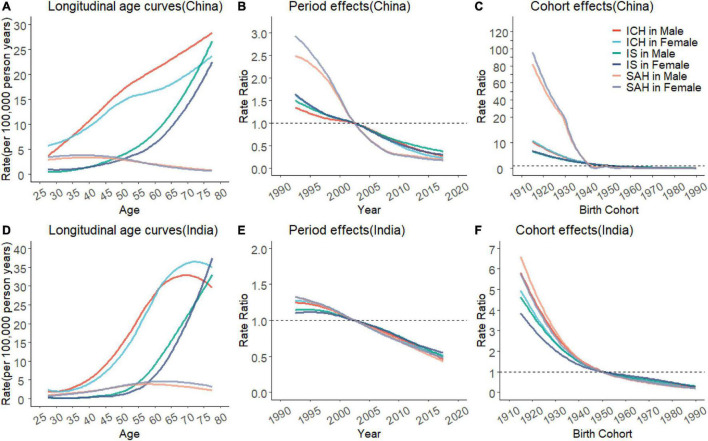
Parameter estimates of age, period, and cohort effects on HAP-attributable subtype stroke mortality rate in China and India from 1990 to 2019. **(A,D)**, fitted longitudinal age curves of HAP-attributable intracerebral hemorrhage (ICH), ischemic stroke (IS), and subarachnoid hemorrhage (SAH) mortality (per 100,000 person-years) in China and India. **(B,E)**, the RRs of each period compared with the reference (2000–2004) adjusted for age and nonlinear cohort effects in China and India. **(C,F)**, the RRs of each cohort compared with the reference (cohort 1950–1954) adjusted for age and nonlinear period effects in China and India.

Figure 3 presents the estimates for age, period, and cohort effects for subtypes of stroke. The risks of HAP-attributable ICH and IS deaths increased markedly with age in China (Figure 3A) and India (Figure 3D), with ICH higher than IS in the same age group; India had the slowest growth for HAP-attributable SAH deaths across all age groups compared with the gradual decline in China. Period effects for HAP-attributable SAH deaths in China dropped steeply with age, implying a noticeable improvement in HAP-attributable SAH mortality (Figure 3B). In contrast, downward trends for stroke subtypes in India kept at almost the same pace (Figure 3E), with equal improvements in HAP-attributable ICH, IS, and SAH mortality. For cohort effects (Figures 3C,F), the most striking reductions (reducing from 91.24 in 1915–1919 to 0.02 in 1990–1994 birth cohorts for males and 108.20–0.01 for females) were seen in HAP-attributable SAH in China. Nevertheless, the drop of HAP-attributable SAH across cohorts in India appeared not much greater than ICH and IS. In brief, compared to China, fewer improvements in mortality of HAP-attributable ICH and IS were gained in India.

For the results of the Wald tests (Table 2), besides all the values of local drifts and net drifts, the period and cohort RRs were statistically significant (*P* < 0.01) for males and females in both the countries.

**TABLE 2:** Statistical parameters for overall- and age-specific annual percent changes in age-period-cohort models.

Diseases	Sex	All local drifts = net drift		Net drifts = 0		All period RR = 1		All cohort RR = 1	
		Wald-tests	*P*-value	Wald-tests	*P*-value	Wald-tests	*P*-value	Wald-tests	*P*-value
**China**									
Stroke	Both	137.3485604	<0.01	8649.532817	<0.01	9013.963929	<0.01	22881.21897	<0.01
ICH	M	72.65870701	<0.01	5939.333484	<0.01	6651.207252	<0.01	14359.03473	<0.01
	F	38.12767925	<0.01	1838.893599	<0.01	2008.893295	<0.01	5106.562975	<0.01
IS	M	124.5439019	<0.01	5159.615002	<0.01	5732.312388	<0.01	34384.30819	<0.01
	F	108.8991544	<0.01	1524.820089	<0.01	1731.674717	<0.01	8879.088998	<0.01
SAH	M	155.2198119	<0.01	8711.955452	<0.01	9970.935547	<0.01	15530.55206	<0.01
	F	49.86183869	<0.01	4438.086611	<0.01	5101.162039	<0.01	9308.21867	<0.01
**India**									
Stroke	Both	71.29796406	<0.01	1677.724833	<0.01	1858.038624	<0.01	3983.693117	<0.01
ICH	M	118.1123193	<0.01	1517.318872	<0.01	1756.05248	<0.01	4052.151654	<0.01
	F	47.75939131	<0.01	630.3353324	<0.01	694.9914619	<0.01	1679.767505	<0.01
IS	M	77.9751195	<0.01	396.8159417	<0.01	691.0998631	<0.01	4390.200821	<0.01
	F	67.11861967	<0.01	197.387432	<0.01	318.7866217	<0.01	2059.51138	<0.01
SAH	M	222.0716851	<0.01	8221.62568	<0.01	8376.434625	<0.01	10100.76599	<0.01
	F	89.74361995	<0.01	2919.571988	<0.01	3018.314219	<0.01	4134.929831	<0.01

## Discussion

This study comprehensively estimated on temporal trends in stroke and subtypes deaths attributable to long-term exposure to HAP in China and India. We found the trends of the ASMRs in stroke in China decreased by 39.8% compared with 35.8% in India, while stroke subtypes in both sexes and countries showed consecutive significant declines from 1990 to 2019. The age-specific and cohort-specific HAP-attributable stroke mortality declined over time in China and India. The substantially increasing age effects were presented for stroke and subtypes from 25 to 84 years. China had a rapid reduction in the independent period and cohort effects. Also, the risk of death for SAH declined the most strikingly for both sexes in period and cohort effects. Reductions of India were less favorable than China, but the independent period and cohort effects progressively decreased during the entire period for both males and females. Our analyses uncovered that the risks of stroke death attributable to HAP have been lessened in China and India. This may be because solid fuels were not a habit of usage for household cooking or heating in recent years, so the danger of death for stroke and subtypes attributable to particulate matter exposure was minimized.

One of the interesting findings was related to the fact that India and China were once highly dependent on fossil fuels which was the dangerous risk factor affecting the health of people. HAP is possible to induce the same or stronger adverse effects on the cerebrovascular system in human subjects compared to outdoor air pollution because humans spend approximately 90% of their time in indoor environments (Klepeis et al., 2001). According to the documented scientific statement from the American Heart Association, the particulate matter released from the combustion of solid flues can trigger acute cardiovascular events, possible biological mechanisms include systemic vascular oxidative stress response, endothelial dysfunction, and plaque formation (Naeher et al., 2007; Brook et al., 2010). In addition, particulate matter can directly penetrate the blood-brain barrier and be translocated into the brain, the potential neurotoxicity cannot be ignored (Mondal et al., 2018; Munzel et al., 2018). More importantly, particles in HAP could be active or have an interaction effect with other risk factors of stroke, such as increasing the incidence of hypertension (Chen et al., 2014; Giorginia et al., 2016).

The combustion of solid fuels for household cooking or heating is the most crucial source of particulate matter exposure. As reported, ambient particulate levels in HAP frequently exceed 100 times the WHO-recommended safety level for sustained periods and account for the majority of personal particulate matter exposure (Warburton et al., 2018). The HAP-attributable stroke mortality increasing with age in China and India might be positively related to more time exposure to HAP and accumulated more harmful particles in the body (Liu et al., 2018; Johnson et al., 2019). Age has been proved as the most important demographic risk factor in stroke, which means stroke burden increases in the elderly (Wang et al., 2017). However, in this study, both the age-specific and cohort-specific HAP-attributable stroke mortality declined since the early period/cohort (Supplementary Tables 1–8). It reflected a transition in epidemiology—the reduction in the risk of premature mortality from HAP-attributable stroke occurs in China and India gradually.

The transition in HAP-attributable stroke mortality is also proved by the period or cohort effects change. China had relatively higher ASMRs than India in the early period. In parallel to the APC analysis, the period and cohort effects in China and India peaked in 1990. Moreover, HAP ranked at 2 in all risk factors attributable to stroke mortality (Supplementary Figure 2) and ranked at 1 in all environmental risk factors attributable to stroke mortality in both China and India (Supplementary Figure 3), which is consistent with studies by the IHME (Roth et al., 2020). As the clean policies continue to advance, initiatives such as replacing solid fuels with liquefied petroleum gas, introducing liquefied petroleum gas into the low-income household, or targeted and innovative subsidies for clean household energy potentially help India achieve the WHO air quality standards within homes (India State-Level Disease Burden Initiative Air Pollution Collaborators, 2019). Likewise, the Chinese National Improved Stove Program and the substitution campaign on household solid fuels substantially mitigate the HAP-attributable stroke burden in recent periods and cohort groups (Edwards et al., 2007). In addition to reduce the use of fossil fuels, the air filtration has been reported that it could be part of a comprehensive strategy to improve indoor air quality and cardiopulmonary health (Chuang et al., 2017). The previous study has reported that houseplants removed particulate air pollution, so the use of houseplants is suggested to be a possible and practical air-purifying system for indoor air quality (Claudio, 2011; Wang et al., 2014). Also, economic improvement has expanded the healthcare coverage and enhanced medical technology. These measures are favorable to control indoor personal particulate matter exposure. In this context, besides the monotonously downward period and cohort effects in India from 1990 to 2019, HAP ranked at 5 in all risk factors attributable to stroke mortality (Supplementary Figure 2) and ranked at 2 in all environmental risk factors attributable to stroke mortality (Supplementary Figure 3) in India (Supplementary Figure 3). Therefore, ranks of HAP were down to 12 in all risk factors (Supplementary Figure 2) and to 2 in all environmental risk factors attributable to stroke mortality (Supplementary Figure 3) in China in 2019. As a result, China and India are on the track to meet the United Nation 2025 targets of a 25% reduction in risk of premature mortality from cardiovascular diseases (WHO, 2013).

Another interesting finding in this study was that despite the proportions of HAP-attributable IS, ICH, and SAH in a total of stroke deaths changed, the HAP-attributable ICH deaths remained the dominant role in the subtypes of stroke from 1990 to 2019. Consistent with other studies (Jiang et al., 2020), the period and cohort effects on HAP-attributable ICH were greater than that on IS, which may be mainly explained by the difference between the mechanism of HAP exposure on ICH and IS. HAP-attributable ICH mortality was linked to blood pressure increasing, oxidative stress promoting, and inflammation, while the HAP-attributable IS mortality was connected to atherosclerosis (Suwa et al., 2002; Araujo, 2011). Albeit, this study denoted the HAP-attributable SAH was relatively rare, it had the highest risk of death among subtypes of stroke in China and India according to period and cohort effects. Unlike India, the HAP-attributable SAH in China proved far more lethal than ICH or IS before 2002 and in those born before 1940. HAP-attributable SAH mortality was associated with blood pressure. Elevated blood pressure is likely to be associated with cerebrovascular rupture, especially for people with high systolic blood pressure (Muller et al., 2019). By reason SAH may contribute to sudden deaths before the patients receive medical attention, it was the most lethal subtype of stroke in the period when stroke management was undeveloped (Feigin et al., 2005).

For sex disparity, the previous study suggested the possibility of a greater risk of death in males with stroke but better survival in females (Roth et al., 2020). However, we found that stroke mortality attributable to HAP was slightly higher in males than females in most age groups and periods. The risk factors of stroke, like alcohol use or tobacco, are more prevalent in males (Fullerton et al., 2008; Meng et al., 2019); these risk factors may have interactive effects on HAP and increase the stroke mortality of males. Yet females spend more time cooking or staying in the kitchen with higher HAP concentrations than other household places (Zhang and Smith, 2007). Given that females tend to be more highly exposed, the sex difference is less pronounced for HAP.

According to the latest World Bank classification (Tian et al., 2018), China has experienced incredibly rapid economic growth and has reached an upper-middle-income level; while India has reached a lower-middle-income level. Though fuels for cooking have declined in India, about 56% of Indians are still exposed to HAP from solid fuels (Hay et al., 2017). Furthermore, marked variations on the frequency of solid fuels use still exist in different states. The underdeveloped areas, especially Bihar, Uttar Pradesh, Rajasthan, and Jharkhand, have the highest level of HAP (Dandona et al., 2017). In China, particularly in western, central, and north-eastern China, most rural residents rely heavily on solid fuels, and clean energy technology has been slow to penetrate (Chen et al., 2018). For these reasons, this study suggested that the uneven HAP exposure in the population of China and India and the existed gaps between recommended goals and the current situation should be called attention on.

The general limitations exist in this study: first, the data used in the study were extracted from the latest GBD study. There are certain deviations in the completeness and accuracy of stroke deaths. Although the GBD 2019 adopts numerous adjustments and corrections to the source, collation, and evaluation of the stroke mortality attribute to HAP to enhance data accuracy and comparability, completely refraining from data inaccuracy thoroughly seemed impossible. Second, in GBD 2019, data of the subdivisional region in China and India are not included. Hence the trends in different provinces, states, or urban and rural areas are not clear. Third, like other studies based on a population level, ecological fallacy might occur because the study might not focus on the individual level. Studies in the future should consider shortages in our research and avoid them.

## Conclusion

Although prominent reductions were observed in HAP-attributable stroke and subtypes mortality during the past 30 years, China and India still suffered uneven HAP-attributable stroke burden. With a rapid reduction in HAP-attributable stroke mortality across all age groups over time and a relatively lower risk of death in recently born cohorts, China on behalf of the successful epidemic transition. Meanwhile, the risk of death for SAH declined the most strikingly for both sexes in period and cohort effects. Progress of India in curbing HAP-attributable stroke burden was less outstanding, but the independent period and cohort effects gradually decreased during the entire period for both sexes. Specifically, males and elder groups are the high-risk populations for stroke mortality attributable to HAP, but mortality in females cannot be ignored, either. Thus, it is of high significance to introduce advanced solid fuels to replace technology and knowledge regarding clean fuel use.

## Data Availability Statement

The datasets presented in this study can be found in online repositories. The names of the repository/repositories and accession number(s) can be found in the article/Supplementary Material. The data set supporting the conclusions of this article is available in the GBD Data Tool repository (http://ghdx.healthdata.org/gbd-results-tool). This study used publicly available deidentified data accessed from the Global Burden of Disease Study 2019 repository.

## Ethics Statement

Ethical review and approval was not required for the study on human participants in accordance with the local legislation and institutional requirements. Written informed consent for participation was not required for this study in accordance with the national legislation and the institutional requirements.

## Author Contributions

CY and YM: conception and design of the study and analysis and/or interpretation of data. YM and DY: collating data and writing the original manuscript. YM: visualization. YM, CY, DY, JB, YZ, and QH: reviewing and editing the manuscript. CY: funding acquisition. All authors read and agreed to the published version of the manuscript.

## Conflict of Interest

The authors declare that the research was conducted in the absence of any commercial or financial relationships that could be construed as a potential conflict of interest.

## Publisher’s Note

All claims expressed in this article are solely those of the authors and do not necessarily represent those of their affiliated organizations, or those of the publisher, the editors and the reviewers. Any product that may be evaluated in this article, or claim that may be made by its manufacturer, is not guaranteed or endorsed by the publisher.

## Footnotes

1
http://ghdx.healthdata.org/gbd-results-tool

2
https://github.com/CBIIT/nci-webtools-dceg-age-period-cohort

## References

[B1] Anderson C. S. Chaturvedi S. (2018). Stroke in China and India: big populations, big challenges. *Neurology* 91 643–644. 10.1212/WNL.0000000000006270 30158162

[B2] Araujo J. A. (2011). Particulate air pollution, systemic oxidative stress, inflammation, and atherosclerosis. *Air Qual. Atmos. Health* 4 79–93. 10.1007/s11869-010-0101-8 21461032 PMC3040314

[B3] Balmes J. R. (2019). Household air pollution from domestic combustion of solid fuels and health. *J. Allergy Clin. Immunol.* 143 1979–1987. 10.1016/j.jaci.2019.04.016 31176380

[B4] Bell A. (2020). Age period cohort analysis: a review of what we should and shouldn’t do. *Ann. Hum. Biol.* 47 208–217. 10.1080/03014460.2019.1707872 32429768

[B5] Brook R. D. Rajagopalan S. Pope C. A. III Brook J. R. Bhatnagar A. Diez-Roux A. V. (2010). Particulate matter air pollution and cardiovascular disease: an update to the scientific statement from the American Heart Association. *Circulation* 121 2331–2378. 10.1161/CIR.0b013e3181dbece1 20458016

[B6] Bruce N. Perez-Padilla R. Albalak R. (2000). Indoor air pollution in developing countries: a major environmental and public health challenge. *Bull. World Health Organ.* 78 1078–1092. 11019457 PMC2560841

[B7] Burnett R. T. Pope C. A. III Ezzati M. Olives C. Lim S. S. Mehta S. (2014). An integrated risk function for estimating the global burden of disease attributable to ambient fine particulate matter exposure. *Environ. Health Perspect.* 122 397–403. 10.1289/ehp.1307049 24518036 PMC3984213

[B8] Burnett R. Chen H. Szyszkowicz M. Fann N. Hubbell B. Pope C. A. III (2018). Global estimates of mortality associated with long-term exposure to outdoor fine particulate matter. *Proc. Natl. Acad. Sci. U.S.A.* 115 9592–9597. 10.1073/pnas.1803222115 30181279 PMC6156628

[B9] Cao J. H. Eshak E. S. Liu K. Y. Gero K. Liu Z. M. Yu C. H. (2019). Age-period-cohort analysis of stroke mortality attributable to high sodium intake in China and Japan GBD data 1990 to 2016. *Stroke* 50 1648–1654. 10.1161/STROKEAHA.118.024617 31195942 PMC6594775

[B10] Chen H. Burnett R. T. Kwong J. C. Villeneuve P. J. Goldberg M. S. Brook R. D. (2014). Spatial association between ambient fine particulate matter and incident hypertension. *Circulation* 129 562–569. 10.1161/CIRCULATIONAHA.113.003532 24190962

[B11] Chen Y. L. Shen H. Z. Smith K. R. Guan D. B. Chen Y. C. Shen G. F. (2018). Estimating household air pollution exposures and health impacts from space heating in rural China. *Environ. Int.* 119 117–124. 10.1016/j.envint.2018.04.054 29957353

[B12] Chuang H. C. Ho K. F. Lin L. Y. Chang T. Y. Hong G. B. Ma C. M. I (2017). Long-term indoor air conditioner filtration and cardiovascular health: a randomized crossover intervention study. *Environ. Int.* 106 91–96. 10.1016/j.envint.2017.06.008 28624750

[B13] Claudio L. (2011). Planting healthier indoor air. *Environ. Health Perspect.* 119 A426–A427. 10.1289/ehp.119-a426 22069776 PMC3230460

[B14] Cohen A. J. Brauer M. Burnett R. Anderson H. R. Frostad J. Estep K. (2017). Estimates and 25-year trends of the global burden of disease attributable to ambient air pollution: an analysis of data from the global burden of diseases study 2015. *Lancet* 389 1907–1918. 10.1016/S0140-6736(17)30505-6 28408086 PMC5439030

[B15] Dandona L. Dandona R. Kumar G. A. Shukla D. Swaminathan S. (2017). Nations within a nation: variations in epidemiological transition across the States of India, 1990-2016 in the Global Burden of Disease Study – supplementary information. *Lancet* 390, 2437–2460. 10.1016/S0140-6736(17)32804-0 29150201 PMC5720596

[B16] Edwards R. D. Li Y. He G. Yin Z. Sinton J. Peabody J. (2007). Household CO and PM measured as part of a review of China’s National improved stove program. *Indoor Air* 17 189–203. 10.1111/j.1600-0668.2007.00465.x 17542832

[B17] Feigin V. L. Rinkel G. J. E. Lawes C. M. M. Algra A. Bennett D. A. van Gijn J. (2005). Risk factors for subarachnoid hemorrhage – an updated systematic review of epidemiological studies. *Stroke* 36 2773–2780. 10.1161/01.STR.0000190838.02954.e8 16282541

[B18] Fullerton D. G. Nigel B. Gordon S. B. (2008). Indoor air pollution from biomass fuel smoke is a major health concern in the developing world. *Trans. R. Soc. Trop. Med. Hyg.* 102 843–851. 10.1016/j.trstmh.2008.05.028 18639310 PMC2568866

[B19] GBD 2015 Mortality and Causes of Death Collaborators (2016). Global, regional, and national life expectancy, all-cause mortality, and cause-specific mortality for 249 causes of death, 1980-2015: a systematic analysis for the Global burden of disease study 2015. *Lancet* 388 1459–1544. 10.1016/S0140-6736(16)31012-1 27733281 PMC5388903

[B20] GBD 2016 Risk Factors Collaborators (2018). Global, regional, and national comparative risk assessment of 84 behavioural, environmental and occupational, and metabolic risks or clusters of risks for 195 countries and territories, 1990-2017: a systematic analysis for the Global Burden of Disease Study 2017. *Lancet* 392 1923–1994. 10.1016/S0140-6736(18)32225-6 30496105 PMC6227755

[B21] GBD 2017 Causes of Death Collaborators (2018). Global, regional, and national age-sex-specific mortality for 282 causes of death in 195 countries and territories, 1980-2017: a systematic analysis for the Global Burden of disease study 2017. *Lancet* 392 1736–1788. 10.1016/S0140-6736(18)32203-7 30496103 PMC6227606

[B22] GBD 2019 Risk Factors Collaborators (2020). Global burden of 87 risk factors in 204 countries and territories, 1990–2019: a systematic analysis for the Global Burden of Disease study 2019. *Lancet* 396, 1223–1249. 10.1016/S0140-6736(20)30752-2 33069327 PMC7566194

[B23] Giorginia P. Di Giosia P. Grassi D. Rubenfire M. Brook R. D. Ferri C. (2016). Air pollution exposure and blood pressure: an updated review of the literature. *Curr. Pharm. Design* 22 28–51. 10.2174/1381612822666151109111712 26548310

[B24] Hay S. I. Abdulkader R. S. Afshin A. Agarwal S. K. Zodpey S. (2017). *India: Health of the Nation’s States – The India State-level Disease Burden Initiative.* New Delhi: Indian Council of Medical Research

[B25] Hisham N. F. Bayraktutan U. (2013). Epidemiology, pathophysiology, and treatment of hypertension in ischaemic stroke patients. *J. Stroke Cerebrovasc.* 22 E4–E14. 10.1016/j.jstrokecerebrovasdis.2012.05.001 22682972

[B26] Hystad P. Duong M. Brauer M. Larkin A. Arku R. Kurmi O. P. (2019). Health effects of household solid fuel use: findings from 11 Countries within the prospective urban and rural epidemiology study. *Environ. Health Perspect.* 127:57003. 10.1289/EHP3915 31067132 PMC6791569

[B27] India State-Level Disease Burden Initiative Air Pollution Collaborators (2019). The impact of air pollution on deaths, disease burden, and life expectancy across the states of India: the Global Burden of disease study 2017. *Lancet Planetary Health* 3 E26–E39. 10.1016/S2542-5196(18)30261-4 30528905 PMC6358127

[B28] Jha P. Gajalakshmi V. Gupta P. C. Kumar R. Mony P. Dhingra N. (2006). Study, Prospective study of one million deaths in India: rationale, design, and validation results. *PLoS Med* 3:e18. 10.1371/journal.pmed.0030018 16354108 PMC1316066

[B29] Jiang Y. F. Lu H. Y. Man Q. H. Liu Z. Q. Wang L. P. Wang Y. Z. (2020). Stroke burden and mortality attributable to ambient fine particulate matter pollution in 195 countries and territories and trend analysis from 1990 to 2017. *Environ. Res.* 184:109327. 10.1016/j.envres.2020.109327 32151843

[B30] Johnson C. O. Nguyen M. Roth G. A. Nichols E. Alam T. Abate D. (2019). Global, regional, and national burden of stroke, 1990-2016: a systematic analysis for the Global Burden of Disease Study 2016. *Lancet Neurol.* 18 439–458. 10.1016/S1474-4422(19)30034-1 30871944 PMC6494974

[B31] Klepeis N. E. Nelson W. C. Ott W. R. Robinson J. P. Tsang A. M. Switzer P. (2001). The National human activity pattern survey (NHAPS): a resource for assessing exposure to environmental pollutants. *J. Expo. Anal. Environ. Epidemiol.* 11 231–252. 10.1038/sj.jea.7500165 11477521

[B32] Liu J. Hou B. D. Ma X. W. Liao H. (2018). Solid fuel use for cooking and its health effects on the elderly in rural China. *Environ. Sci. Pollut. R* 25 3669–3680. 10.1007/s11356-017-0720-9 29164467

[B33] Liu Z. Hystad P. Zhang Y. Rangarajan S. Yin L. Wang Y. (2021). Associations of household solid fuel for heating and cooking with hypertension in Chinese adults. *J. Hypertens.* 39 667–676. 10.1097/HJH.0000000000002689 33186328

[B34] Lu H. Tan Z. Liu Z. Wang L. Wang Y. Suo C. (2021). Spatiotemporal trends in stroke burden and mortality attributable to household air pollution from solid fuels in 204 countries and territories from 1990 to 2019. *Sci. Total Environ.* 775:145839. 10.1016/j.scitotenv.2021.145839 33631580

[B35] Meng W. J. Zhong Q. R. Chen Y. L. Shen H. Z. Yun X. Smith K. R. (2019). Energy and air pollution benefits of household fuel policies in northern China. *Proc. Natl. Acad. Sci. U.S.A.* 116 16773–16780. 10.1073/pnas.1904182116 31383761 PMC6708357

[B36] Mondal N. K. Saha H. Mukherjee B. Tyagi N. Ray M. R. (2018). Inflammation, oxidative stress, and higher expression levels of Nrf2 and NQO1 proteins in the airways of women chronically exposed to biomass fuel smoke. *Mol. Cell Biochem.* 447 63–76. 10.1007/s11010-018-3293-0 29363060

[B37] Muller T. B. Vik A. Romundstad P. R. Sandvei M. S. (2019). Risk factors for unruptured intracranial aneurysms and subarachnoid hemorrhage in a prospective population-based study. *Stroke* 50 2952–2955. 10.1161/STROKEAHA.119.025951 31370767

[B38] Munzel T. Gori T. Al-Kindi S. Deanfield J. Lelieveld J. Daiber A. (2018). Effects of gaseous and solid constituents of air pollution on endothelial function. *Eur. Heart J.* 39 3543–3550. 10.1093/eurheartj/ehy481 30124840 PMC6174028

[B39] Naeher L. P. Brauer M. Lipsett M. Zelikoff J. T. Simpson C. D. Koenig J. Q. (2007). Woodsmoke health effects: a review. *Inhal. Toxicol.* 19 67–106. 10.1080/08958370600985875 17127644

[B40] Robertson C. Gandini S. Boyle P. (1999). Age-period-cohort models: a comparative study of available methodologies. *J. Clin. Epidemiol.* 52 569–583. 10.1016/s0895-4356(99)00033-5 10408997

[B41] Rosenberg P. S. Anderson W. F. (2011). Age-period-cohort models in cancer surveillance research: ready for prime time? *Cancer Epidemiol. Biomarkers Prev.* 20 1263–1268. 10.1158/1055-9965.EPI-11-0421 21610223 PMC3132831

[B42] Rosenberg P. S. Check D. P. Anderson W. F. (2014). A web tool for age-period-cohort analysis of cancer incidence and mortality rates. *Cancer Epidemiol. Biomarkers Prev.* 23 2296–2302. 10.1158/1055-9965.EPI-14-0300 25146089 PMC4221491

[B43] Roth G. A. Mensah G. A. Johnson C. O. Addolorato G. Ammirati E. Baddour L. M. (2020). Global burden of cardiovascular diseases and risk factors, 1990-2019: update from the GBD 2019 study. *J. Am. Coll. Cardiol.* 76 2982–3021. 10.1016/j.jacc.2020.11.010 33309175 PMC7755038

[B44] Rudd K. E. Johnson S. C. Agesa K. M. Shackelford K. A. Tsoi D. Kievlan D. R. (2020). Global, regional, and national sepsis incidence and mortality, 1990-2017: analysis for the Global burden of disease study. *Lancet* 395 200–211. 10.1016/S0140-6736(19)32989-7 31954465 PMC6970225

[B45] Suwa T. Hogg J. C. Quinlan K. B. Ohgami A. Vincent R. van Eeden S. F. (2002). Particulate air pollution induces progression of atherosclerosis. *J. Am. Coll. Cardiol.* 39 935–942. 10.1016/s0735-1097(02)01715-1 11897432

[B46] Tian D. Y. Yang Q. Dong Q. Li N. Yan B. Fan D. S. (2018). Trends in stroke subtypes and vascular risk factors in a stroke center in China over 10 years. *Sci. Rep.* 8:5037. 10.1038/s41598-018-23356-9 29567985 PMC5864718

[B47] Wang Z. K. Hu S. B. Sang S. P. Luo L. S. Yu C. H. (2017). Age-period-cohort analysis of stroke mortality in china: data from the Global burden of disease study 2013. *Stroke* 48 271–275. 10.1161/STROKEAHA.116.015031 27965429

[B48] Wang Z. Q. Pei J. J. Zhang J. S. (2014). Experimental investigation of the formaldehyde removal mechanisms in a dynamic botanical filtration system for indoor air purification. *J. Hazard Mater.* 280 235–243. 10.1016/j.jhazmat.2014.07.059 25164387

[B49] Warburton D. Warburton N. Wigfall C. Chimedsuren O. Lodoisamba D. Lodoysamba S. (2018). Impact of seasonal winter air pollution on health across the lifespan in mongolia and some putative solutions. *Ann. Am. Thorac. Soc.* 15 S86–S90. 10.1513/AnnalsATS.201710-758MG 29676634 PMC6850795

[B50] WHO (2013). *Draft Action Plan for the Prevention and Control of Noncommunicable Diseases 2013–2020.* Rugby: IChemE.

[B51] Xue T. Zhu T. Zheng Y. Liu J. Li X. Zhang Q. (2019). Change in the number of PM2.5-attributed deaths in China from 2000 to 2010: comparison between estimations from census-based epidemiology and pre-established exposure-response functions. *Environ. Int.* 129 430–437. 10.1016/j.envint.2019.05.067 31154145

[B52] Yang Y. Schulhofer-Wohl S. Fu W. J. J. Land K. C. (2008). The intrinsic estimator for age-period-cohort analysis: what it is and how to use it. *Am. J. Sociol.* 113 1697–1736. 10.1086/587154

[B53] Yasin Y. J. Banoub J. A. M. Kanchan T. (2019). GBD 2017 risk factor collaborators. Global, regional, and national comparative risk assessment of 84 behavioural, environmental and occupational, and metabolic risks or clusters of risks for 195 countries and territories, 1990- 2017: a systematic analysis for the Global burden of disease study 2017 (vol 392, pg 1923, 2017). *Lancet* 393 E44–E44. 10.1016/S0140-6736(18)32225-6 PMC6227755 30496105

[B54] Yu K. Lv J. Qiu G. K. Yu C. Q. Guo Y. Bian Z. (2020). Cooking fuels and risk of all-cause and cardiopulmonary mortality in urban China: a prospective cohort study. *Lancet Glob. Health* 8 E430–E439. 10.1016/S2214-109X(19)30525-X 31972151 PMC7031698

[B55] Yu K. Qiu G. K. Chan K. H. Lam K. B. H. Kurmi O. P. Bennett D. A. (2018). Association of solid fuel use with risk of cardiovascular and all-cause mortality in rural China. *J. Am. Med. Assoc.* 319 1351–1361. 10.1001/jama.2018.2151 29614179 PMC5933384

[B56] Zhang J. J. Smith K. R. (2007). Household air pollution from coal and biomass fuels in China: measurements, health impacts, and interventions. *Environ. Health Perspect.* 115 848–855. 10.1289/ehp.9479 17589590 PMC1892127

[B57] Zhou M. G. Wang H. D. Zhu J. Chen W. Q. Wang L. H. Liu S. W. (2016). Cause-specific mortality for 240 causes in China during 1990-2013: a systematic subnational analysis for the Global Burden of Disease Study 2013. *Lancet* 387 251–272. 10.1016/S0140-6736(15)00551-6 26510778

