# Supplementary material for: Time Trends in Stroke and Subtypes Mortality Attributable to Household Air Pollution in Chinese and Indian Adults: An Age-Period-Cohort Analysis Using the Global Burden of Disease Study 2019
Source: Front Aging Neurosci. 2022 Feb 18;14:740549. doi: 10.3389/fnagi.2022.740549 (PMC8895296; doi:10.3389/fnagi.2022.740549)
Supplement: Supplementary file 1 [file Data_Sheet_1.docx]

Supplementary Material

# Supplementary Figures


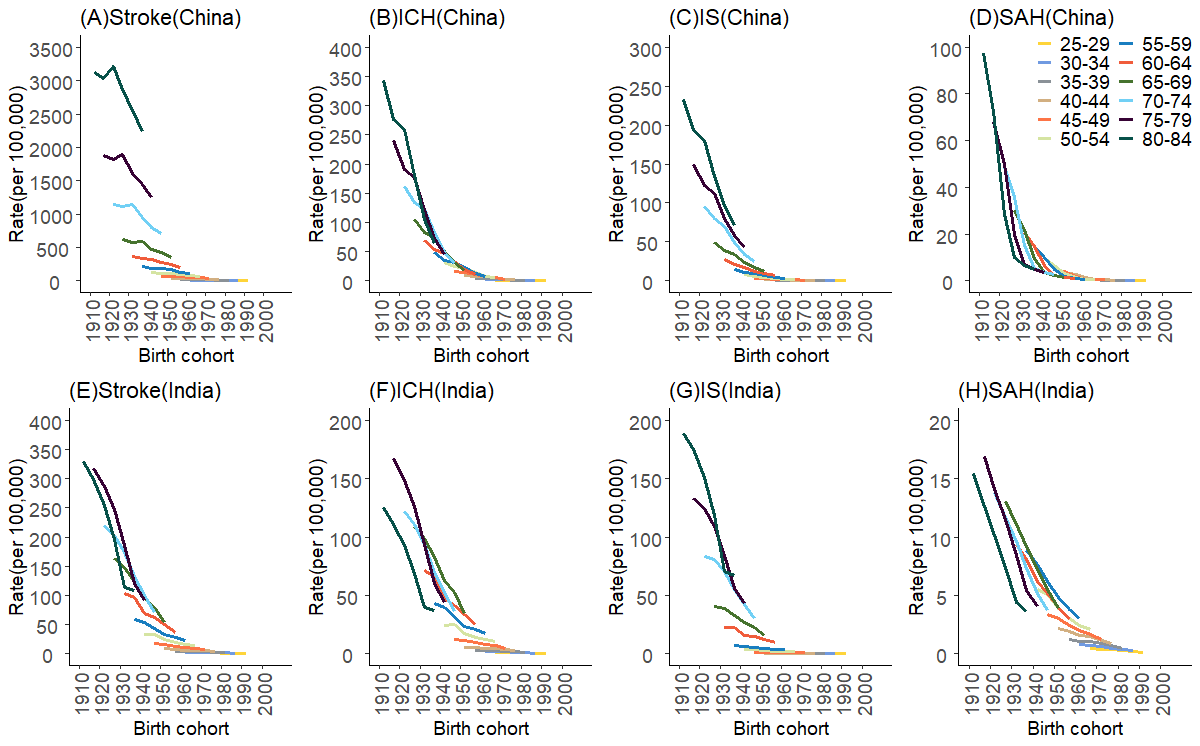


Figure A1. The cohort-specific mortality of HAP-attributable stroke and subtypes in China and India from 1990 to 2019.

A through H, The HAP-attributable stroke mortality data in China and India were arranged into 17 consecutive birth cohorts, including those born from 1915 to 1919 (median, 1917) to 1990 to 1994 (median, 1992), and successive 5-year age intervals from 25 to 29 years (median, 27 years) to 80 to 84 years (median, 82 years) of age (P<0.01 for all).


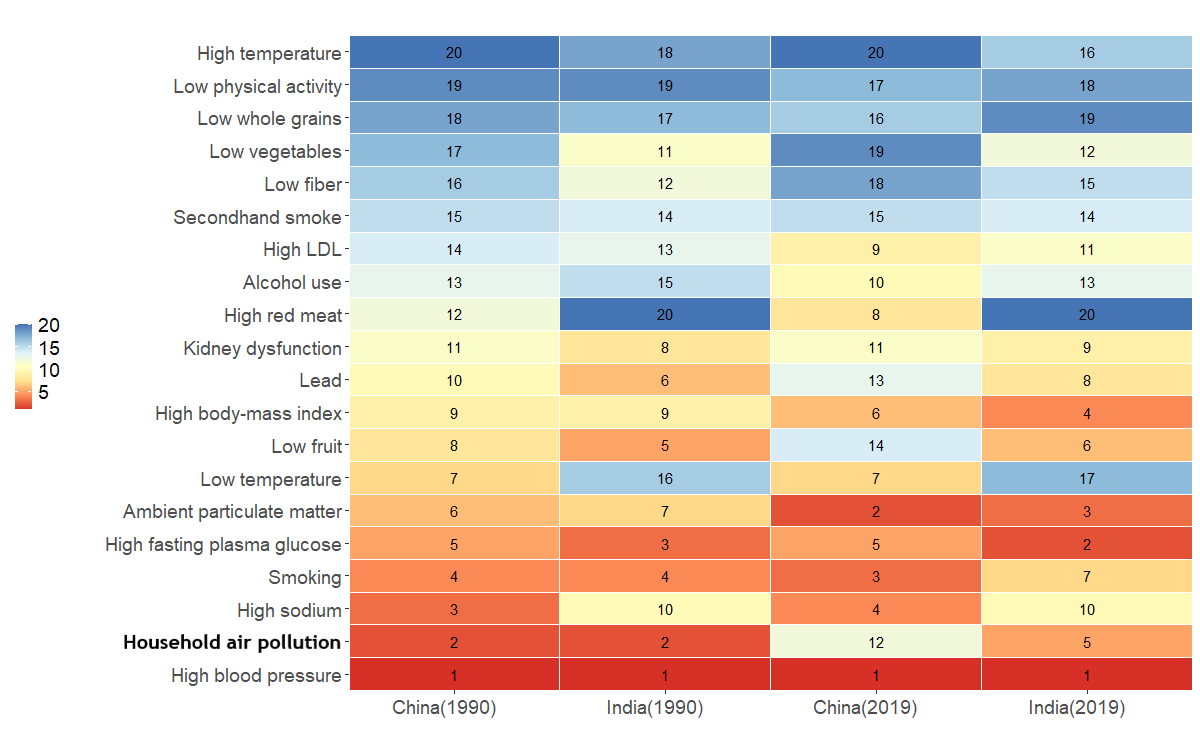


Figure A2. The rank of all risk factors for stroke in China and Indian in 1990 and 2019.


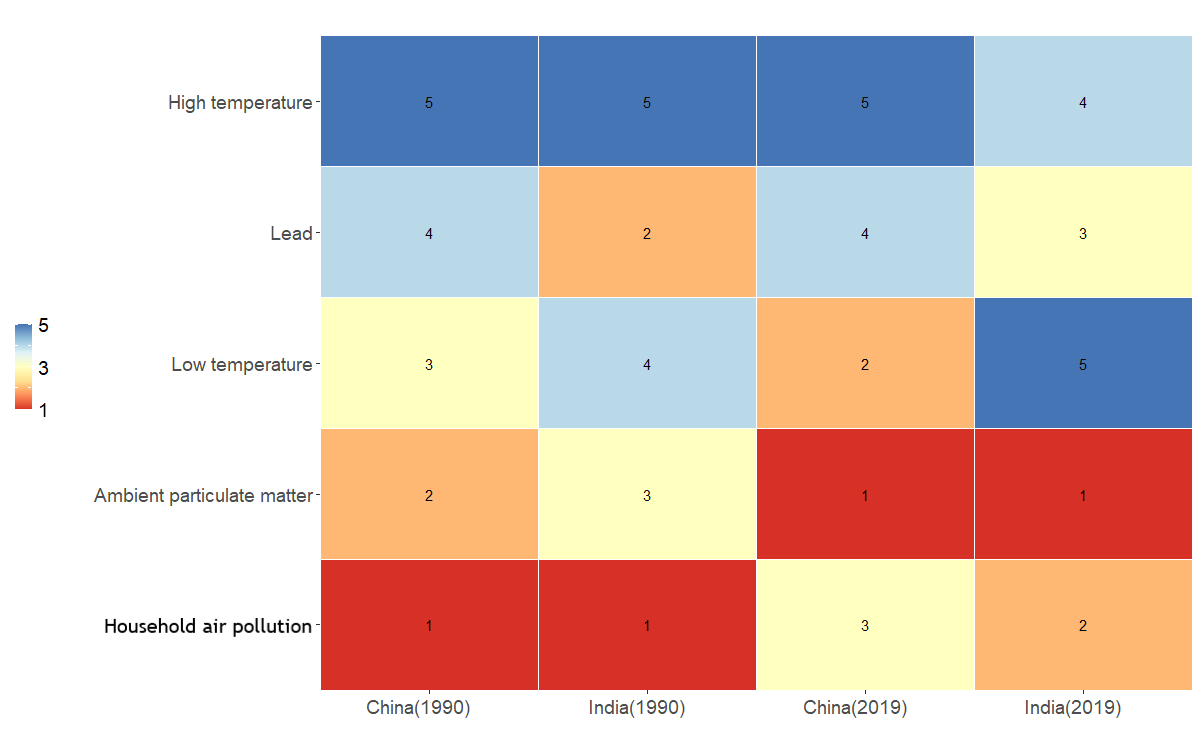


Figure A3. The rank of environmental risk factors for stroke in China and India in 1990 and 2019.

# Supplementary Tables

Table A1. Mortality rate of HAP-attributable stroke by age, period, and median birth cohorts in China, 1990 to 2019 (per 100 000)

| **Median period** | **Age group** | | | | | | | | | | | | **Median birth cohort** |
| --- | --- | --- | --- | --- | --- | --- | --- | --- | --- | --- | --- | --- | --- |
|  | 25-29 | 30-34 | 35-39 | 40-44 | 45-49 | 50-54 | 55-59 | 60-64 | 65-69 | 70-74 | 75-79 | 80-84 |  |
| Stroke(China) |  |  |  |  |  |  |  |  |  |  |  | 673.61 | 1912 |
|  |  |  |  |  |  |  |  |  |  |  | 458.17 | 543.27 | 1917 |
|  |  |  |  |  |  |  |  |  |  | 306.09 | 363.38 | 467.76 | 1922 |
|  |  |  |  |  |  |  |  |  | 183.11 | 249.29 | 308.23 | 323.38 | 1927 |
|  |  |  |  |  |  |  |  | 116.1 | 142.57 | 207.57 | 206.4 | 208.82 | 1932 |
|  |  |  |  |  |  |  | 77.98 | 88.52 | 118.26 | 136.46 | 135.15 | 139.59 | 1937 |
|  |  |  |  |  |  | 48.71 | 54.66 | 71.17 | 76.8 | 86.37 | 92.21 |  | 1942 |
|  |  |  |  |  | 24.87 | 36.63 | 44.41 | 47.02 | 50.13 | 57.83 |  |  | 1947 |
|  |  |  |  | 14.59 | 21.15 | 28.57 | 30.14 | 29.79 | 31.69 |  |  |  | 1952 |
|  |  |  | 6.93 | 11.61 | 15.37 | 18.38 | 16.82 | 18.57 |  |  |  |  | 1957 |
|  |  | 3.45 | 5.61 | 9.2 | 8.94 | 10.09 | 10.94 |  |  |  |  |  | 1962 |
|  | 2.02 | 3.28 | 4.5 | 6.62 | 6.75 | 6.99 |  |  |  |  |  |  | 1967 |
| 1992 | 1.92 | 2.38 | 2.96 | 3.62 | 4.09 |  |  |  |  |  |  |  | 1972 |
| 1997 | 1.3 | 1.35 | 1.75 | 2.63 |  |  |  |  |  |  |  |  | 1977 |
| 2002 | 0.71 | 0.99 | 1.35 |  |  |  |  |  |  |  |  |  | 1982 |
| 2007 | 0.57 | 0.72 |  |  |  |  |  |  |  |  |  |  | 1987 |
| 2012 | 0.4 |  |  |  |  |  |  |  |  |  |  |  | 1992 |
| 2017 |  |  |  |  |  |  |  |  |  |  |  |  |  |

Table A2. Mortality rate of HAP-attributable ICH by age, period, and median birth cohorts in China, 1990 to 2019 (per 100 000)

| **Median period** | **Age group** | | | | | | | | | | | | **Median birth cohort** |
| --- | --- | --- | --- | --- | --- | --- | --- | --- | --- | --- | --- | --- | --- |
|  | 25-29 | 30-34 | 35-39 | 40-44 | 45-49 | 50-54 | 55-59 | 60-64 | 65-69 | 70-74 | 75-79 | 80-84 |  |
| ICH(China) |  |  |  |  |  |  |  |  |  |  |  | 343.74 | 1912 |
|  |  |  |  |  |  |  |  |  |  |  | 241.15 | 275.72 | 1917 |
|  |  |  |  |  |  |  |  |  |  | 162.33 | 190.79 | 258.59 | 1922 |
|  |  |  |  |  |  |  |  |  | 104.51 | 133.93 | 176.67 | 179.6 | 1927 |
|  |  |  |  |  |  |  |  | 68.52 | 82.26 | 122.17 | 119.66 | 105.63 | 1932 |
|  |  |  |  |  |  |  | 49.15 | 52.79 | 74.77 | 82.05 | 72.63 | 63.38 | 1937 |
|  |  |  |  |  |  | 31.55 | 34.65 | 46.92 | 50.4 | 49.18 | 45.75 |  | 1942 |
|  |  |  |  |  | 16.59 | 24.35 | 30.95 | 32.43 | 31.2 | 30.41 |  |  | 1947 |
|  |  |  |  | 9.51 | 14.38 | 20.8 | 21.99 | 19.47 | 18.13 |  |  |  | 1952 |
|  |  |  | 4.56 | 7.79 | 11.42 | 13.89 | 11.79 | 11.36 |  |  |  |  | 1957 |
|  |  | 2.26 | 3.82 | 6.84 | 6.84 | 7.34 | 7.23 |  |  |  |  |  | 1962 |
|  | 1.31 | 2.21 | 3.35 | 5.19 | 5.12 | 4.89 |  |  |  |  |  |  | 1967 |
| 1992 | 1.28 | 1.74 | 2.3 | 2.76 | 2.98 |  |  |  |  |  |  |  | 1972 |
| 1997 | 0.93 | 1.02 | 1.33 | 1.95 |  |  |  |  |  |  |  |  | 1977 |
| 2002 | 0.53 | 0.76 | 1.02 |  |  |  |  |  |  |  |  |  | 1982 |
| 2007 | 0.43 | 0.54 |  |  |  |  |  |  |  |  |  |  | 1987 |
| 2012 | 0.3 |  |  |  |  |  |  |  |  |  |  |  | 1992 |
| 2017 |  |  |  |  |  |  |  |  |  |  |  |  |  |

Table A3. Mortality rate of HAP-attributable IS by age, period, and median birth cohorts in China, 1990 to 2019 (per 100 000)

| **Median period** | **Age group** | | | | | | | | | | | | **Median birth cohort** |
| --- | --- | --- | --- | --- | --- | --- | --- | --- | --- | --- | --- | --- | --- |
|  | 25-29 | 30-34 | 35-39 | 40-44 | 45-49 | 50-54 | 55-59 | 60-64 | 65-69 | 70-74 | 75-79 | 80-84 |  |
| IS(China) |  |  |  |  |  |  |  |  |  |  |  | 232.46 | 1912 |
|  |  |  |  |  |  |  |  |  |  |  | 148.78 | 194.94 | 1917 |
|  |  |  |  |  |  |  |  |  |  | 95.02 | 122.89 | 180.63 | 1922 |
|  |  |  |  |  |  |  |  |  | 48.65 | 79.54 | 111.61 | 133.93 | 1927 |
|  |  |  |  |  |  |  |  | 27.05 | 38.77 | 70.24 | 79.87 | 96.68 | 1932 |
|  |  |  |  |  |  |  | 14.22 | 21.14 | 34.03 | 49.09 | 57.84 | 71.43 | 1937 |
|  |  |  |  |  |  | 7.55 | 10.04 | 17.69 | 22.95 | 33.67 | 42.84 |  | 1942 |
|  |  |  |  |  | 3.32 | 5.71 | 8.73 | 12.24 | 16.64 | 24.88 |  |  | 1947 |
|  |  |  |  | 1.65 | 2.88 | 4.76 | 6.35 | 8.8 | 11.95 |  |  |  | 1952 |
|  |  |  | 0.7 | 1.31 | 2.25 | 3.26 | 3.96 | 6.16 |  |  |  |  | 1957 |
|  |  | 0.36 | 0.58 | 1.1 | 1.38 | 2.02 | 2.95 |  |  |  |  |  | 1962 |
|  | 0.2 | 0.34 | 0.5 | 0.83 | 1.11 | 1.56 |  |  |  |  |  |  | 1967 |
| 1992 | 0.2 | 0.26 | 0.34 | 0.49 | 0.75 |  |  |  |  |  |  |  | 1972 |
| 1997 | 0.14 | 0.15 | 0.2 | 0.4 |  |  |  |  |  |  |  |  | 1977 |
| 2002 | 0.07 | 0.11 | 0.17 |  |  |  |  |  |  |  |  |  | 1982 |
| 2007 | 0.06 | 0.08 |  |  |  |  |  |  |  |  |  |  | 1987 |
| 2012 | 0.04 |  |  |  |  |  |  |  |  |  |  |  | 1992 |
| 2017 |  |  |  |  |  |  |  |  |  |  |  |  |  |

Table A4. Mortality rate of HAP-attributable SAH by age, period, and median birth cohorts in China, 1990 to 2019 (per 100 000)

| **Median period** | **Age group** | | | | | | | | | | | | **Median birth cohort** |
| --- | --- | --- | --- | --- | --- | --- | --- | --- | --- | --- | --- | --- | --- |
|  | 25-29 | 30-34 | 35-39 | 40-44 | 45-49 | 50-54 | 55-59 | 60-64 | 65-69 | 70-74 | 75-79 | 80-84 |  |
| SAH(China) |  |  |  |  |  |  |  |  |  |  |  | 97.41 | 1912 |
|  |  |  |  |  |  |  |  |  |  |  | 68.24 | 72.62 | 1917 |
|  |  |  |  |  |  |  |  |  |  | 48.75 | 49.7 | 28.54 | 1922 |
|  |  |  |  |  |  |  |  |  | 29.96 | 35.82 | 19.95 | 9.85 | 1927 |
|  |  |  |  |  |  |  |  | 20.53 | 21.54 | 15.16 | 6.87 | 6.5 | 1932 |
|  |  |  |  |  |  |  | 14.61 | 14.58 | 9.46 | 5.31 | 4.69 | 4.78 | 1937 |
|  |  |  |  |  |  | 9.61 | 9.97 | 6.56 | 3.45 | 3.52 | 3.62 |  | 1942 |
|  |  |  |  |  | 4.96 | 6.56 | 4.73 | 2.34 | 2.29 | 2.54 |  |  | 1947 |
|  |  |  |  | 3.42 | 3.89 | 3.01 | 1.79 | 1.51 | 1.6 |  |  |  | 1952 |
|  |  |  | 1.67 | 2.5 | 1.7 | 1.23 | 1.07 | 1.05 |  |  |  |  | 1957 |
|  |  | 0.83 | 1.22 | 1.27 | 0.71 | 0.73 | 0.76 |  |  |  |  |  | 1962 |
|  | 0.5 | 0.72 | 0.65 | 0.61 | 0.53 | 0.55 |  |  |  |  |  |  | 1967 |
| 1992 | 0.43 | 0.38 | 0.33 | 0.37 | 0.37 |  |  |  |  |  |  |  | 1972 |
| 1997 | 0.23 | 0.18 | 0.21 | 0.28 |  |  |  |  |  |  |  |  | 1977 |
| 2002 | 0.1 | 0.12 | 0.16 |  |  |  |  |  |  |  |  |  | 1982 |
| 2007 | 0.08 | 0.09 |  |  |  |  |  |  |  |  |  |  | 1987 |
| 2012 | 0.07 |  |  |  |  |  |  |  |  |  |  |  | 1992 |
| 2017 |  |  |  |  |  |  |  |  |  |  |  |  |  |

Table A5. Mortality rate of HAP-attributable stroke by age, period, and median birth cohorts in India, 1990 to 2019 (per 100 000)

| **Median period** | **Age group** | | | | | | | | | | | | **Median birth cohort** |
| --- | --- | --- | --- | --- | --- | --- | --- | --- | --- | --- | --- | --- | --- |
|  | 25-29 | 30-34 | 35-39 | 40-44 | 45-49 | 50-54 | 55-59 | 60-64 | 65-69 | 70-74 | 75-79 | 80-84 |  |
| Stroke(India) |  |  |  |  |  |  |  |  |  |  |  | 329.79 | 1912 |
|  |  |  |  |  |  |  |  |  |  |  | 317.2 | 298.4 | 1917 |
|  |  |  |  |  |  |  |  |  |  | 219.67 | 286.61 | 253.48 | 1922 |
|  |  |  |  |  |  |  |  |  | 163.16 | 203.15 | 246.97 | 195.04 | 1927 |
|  |  |  |  |  |  |  |  | 103.78 | 148.13 | 173.25 | 186.33 | 113.83 | 1932 |
|  |  |  |  |  |  |  | 58.36 | 96.75 | 124.6 | 131.73 | 120.92 | 107.81 | 1937 |
|  |  |  |  |  |  | 32.73 | 53.53 | 69.42 | 96.68 | 99.71 | 91.26 |  | 1942 |
|  |  |  |  |  | 17.09 | 33.4 | 43.49 | 61.67 | 79.77 | 70.79 |  |  | 1947 |
|  |  |  |  | 8.31 | 16.03 | 23.74 | 32.6 | 49.59 | 53.92 |  |  |  | 1952 |
|  |  |  | 4.39 | 7.96 | 12.96 | 18.99 | 28.72 | 36.94 |  |  |  |  | 1957 |
|  |  | 2.78 | 4.05 | 6.45 | 10.96 | 16.3 | 23.54 |  |  |  |  |  | 1962 |
|  | 1.55 | 2.74 | 4.12 | 6.18 | 9.61 | 14.4 |  |  |  |  |  |  | 1967 |
| 1992 | 1.57 | 2.31 | 3.46 | 4.97 | 6.75 |  |  |  |  |  |  |  | 1972 |
| 1997 | 1.48 | 1.93 | 2.91 | 3.83 |  |  |  |  |  |  |  |  | 1977 |
| 2002 | 1.08 | 1.43 | 1.97 |  |  |  |  |  |  |  |  |  | 1982 |
| 2007 | 0.83 | 1.02 |  |  |  |  |  |  |  |  |  |  | 1987 |
| 2012 | 0.51 |  |  |  |  |  |  |  |  |  |  |  | 1992 |
| 2017 |  |  |  |  |  |  |  |  |  |  |  |  |  |

Table A6. Mortality rate of HAP-attributable ICH by age, period, and median birth cohorts in India, 1990 to 2019 (per 100 000)

| **Median period** | **Age group** | | | | | | | | | | | | **Median birth cohort** |
| --- | --- | --- | --- | --- | --- | --- | --- | --- | --- | --- | --- | --- | --- |
|  | 25-29 | 30-34 | 35-39 | 40-44 | 45-49 | 50-54 | 55-59 | 60-64 | 65-69 | 70-74 | 75-79 | 80-84 |  |
| ICH(India) |  |  |  |  |  |  |  |  |  |  |  | 125.53 | 1912 |
|  |  |  |  |  |  |  |  |  |  |  | 166.92 | 110.73 | 1917 |
|  |  |  |  |  |  |  |  |  |  | 122.12 | 148.5 | 92.52 | 1922 |
|  |  |  |  |  |  |  |  |  | 109.35 | 110.87 | 126.09 | 69.16 | 1927 |
|  |  |  |  |  |  |  |  | 71.51 | 98.35 | 93.26 | 93.44 | 39.35 | 1932 |
|  |  |  |  |  |  |  | 42.75 | 66.49 | 82.35 | 69.59 | 59.87 | 36.8 | 1937 |
|  |  |  |  |  |  | 23.95 | 39.33 | 47.19 | 63.1 | 52.4 | 44.33 |  | 1942 |
|  |  |  |  |  | 12.27 | 24.79 | 31.82 | 41.79 | 52.12 | 36.52 |  |  | 1947 |
|  |  |  |  | 5.58 | 11.6 | 17.34 | 23.55 | 33.59 | 34.71 |  |  |  | 1952 |
|  |  |  | 2.88 | 5.46 | 9.33 | 13.83 | 20.99 | 24.75 |  |  |  |  | 1957 |
|  |  | 1.82 | 2.7 | 4.4 | 7.89 | 11.96 | 17.22 |  |  |  |  |  | 1962 |
|  | 1.01 | 1.83 | 2.8 | 4.27 | 7 | 10.65 |  |  |  |  |  |  | 1967 |
| 1992 | 1.04 | 1.54 | 2.35 | 3.44 | 4.86 |  |  |  |  |  |  |  | 1972 |
| 1997 | 0.98 | 1.29 | 1.99 | 2.62 |  |  |  |  |  |  |  |  | 1977 |
| 2002 | 0.71 | 0.95 | 1.33 |  |  |  |  |  |  |  |  |  | 1982 |
| 2007 | 0.54 | 0.67 |  |  |  |  |  |  |  |  |  |  | 1987 |
| 2012 | 0.32 |  |  |  |  |  |  |  |  |  |  |  | 1992 |
| 2017 |  |  |  |  |  |  |  |  |  |  |  |  |  |

Table A7. Mortality rate of HAP-attributable IS by age, period, and median birth cohorts in India, 1990 to 2019 (per 100 000)

| **Median period** | **Age group** | | | | | | | | | | | | **Median birth cohort** |
| --- | --- | --- | --- | --- | --- | --- | --- | --- | --- | --- | --- | --- | --- |
|  | 25-29 | 30-34 | 35-39 | 40-44 | 45-49 | 50-54 | 55-59 | 60-64 | 65-69 | 70-74 | 75-79 | 80-84 |  |
| IS(India) |  |  |  |  |  |  |  |  |  |  |  | 188.85 | 1912 |
|  |  |  |  |  |  |  |  |  |  |  | 133.39 | 175.04 | 1917 |
|  |  |  |  |  |  |  |  |  |  | 83.72 | 124.06 | 150.8 | 1922 |
|  |  |  |  |  |  |  |  |  | 40.73 | 80.61 | 109.37 | 118.46 | 1927 |
|  |  |  |  |  |  |  |  | 22.94 | 38.7 | 70.33 | 84.46 | 70.11 | 1932 |
|  |  |  |  |  |  |  | 6.79 | 22.22 | 33.03 | 54.86 | 55.61 | 67.41 | 1937 |
|  |  |  |  |  |  | 3.3 | 6.54 | 16.05 | 26.4 | 42.17 | 42.9 |  | 1942 |
|  |  |  |  |  | 1.46 | 3.55 | 5.46 | 14.73 | 22.17 | 30.59 |  |  | 1947 |
|  |  |  |  | 0.56 | 1.43 | 2.55 | 4.25 | 12.01 | 15.36 |  |  |  | 1952 |
|  |  |  | 0.29 | 0.56 | 1.19 | 2.14 | 3.92 | 9.26 |  |  |  |  | 1957 |
|  |  | 0.2 | 0.28 | 0.46 | 1.05 | 1.88 | 3.32 |  |  |  |  |  | 1962 |
|  | 0.12 | 0.21 | 0.3 | 0.47 | 0.95 | 1.69 |  |  |  |  |  |  | 1967 |
| 1992 | 0.12 | 0.18 | 0.26 | 0.38 | 0.68 |  |  |  |  |  |  |  | 1972 |
| 1997 | 0.12 | 0.15 | 0.22 | 0.31 |  |  |  |  |  |  |  |  | 1977 |
| 2002 | 0.09 | 0.11 | 0.16 |  |  |  |  |  |  |  |  |  | 1982 |
| 2007 | 0.07 | 0.08 |  |  |  |  |  |  |  |  |  |  | 1987 |
| 2012 | 0.04 |  |  |  |  |  |  |  |  |  |  |  | 1992 |
| 2017 |  |  |  |  |  |  |  |  |  |  |  |  |  |

Table A8. Mortality rate of HAP-attributable SAH by age, period, and median birth cohorts in India, 1990 to 2019 (per 100 000)

| **Median period** | **Age group** | | | | | | | | | | | | **Median birth cohort** |
| --- | --- | --- | --- | --- | --- | --- | --- | --- | --- | --- | --- | --- | --- |
|  | 25-29 | 30-34 | 35-39 | 40-44 | 45-49 | 50-54 | 55-59 | 60-64 | 65-69 | 70-74 | 75-79 | 80-84 |  |
| SAH(India) |  |  |  |  |  |  |  |  |  |  |  | 15.41 | 1912 |
|  |  |  |  |  |  |  |  |  |  |  | 16.9 | 12.63 | 1917 |
|  |  |  |  |  |  |  |  |  |  | 13.83 | 14.05 | 10.15 | 1922 |
|  |  |  |  |  |  |  |  |  | 13.08 | 11.67 | 11.5 | 7.42 | 1927 |
|  |  |  |  |  |  |  |  | 9.32 | 11.08 | 9.66 | 8.43 | 4.36 | 1932 |
|  |  |  |  |  |  |  | 8.82 | 8.04 | 9.23 | 7.28 | 5.45 | 3.6 | 1937 |
|  |  |  |  |  |  | 5.48 | 7.66 | 6.19 | 7.18 | 5.14 | 4.03 |  | 1942 |
|  |  |  |  |  | 3.36 | 5.06 | 6.22 | 5.15 | 5.48 | 3.69 |  |  | 1947 |
|  |  |  |  | 2.17 | 3 | 3.84 | 4.81 | 3.99 | 3.85 |  |  |  | 1952 |
|  |  |  | 1.21 | 1.93 | 2.45 | 3.03 | 3.81 | 2.93 |  |  |  |  | 1957 |
|  |  | 0.76 | 1.07 | 1.59 | 2.01 | 2.46 | 3 |  |  |  |  |  | 1962 |
|  | 0.43 | 0.7 | 1.02 | 1.44 | 1.67 | 2.07 |  |  |  |  |  |  | 1967 |
| 1992 | 0.41 | 0.59 | 0.86 | 1.15 | 1.22 |  |  |  |  |  |  |  | 1972 |
| 1997 | 0.37 | 0.49 | 0.7 | 0.9 |  |  |  |  |  |  |  |  | 1977 |
| 2002 | 0.29 | 0.36 | 0.49 |  |  |  |  |  |  |  |  |  | 1982 |
| 2007 | 0.22 | 0.27 |  |  |  |  |  |  |  |  |  |  | 1987 |
| 2012 | 0.15 |  |  |  |  |  |  |  |  |  |  |  | 1992 |
| 2017 |  |  |  |  |  |  |  |  |  |  |  |  |  |
